# Pediatric in-hospital life-threatening emergencies and cardiac arrest in France: adherence to international guidelines and barriers to implementation

**DOI:** 10.1016/j.resplu.2026.101261

**Published:** 2026-02-10

**Authors:** Marguerite Lockhart-Bouron, Johann Exbrayat, Valentine Baert, Hervé Hubert, Morgan Recher, Stéphane Leteurtre, Groupe Francophone de Réanimation et d’Urgence Pédiatrique (GFRUP), Breinig Sophie, Cantais Aymeric, Lun Thomas, Debacker Pauline, Mortamet Guillaume, Morin Luc, Boussicault Gérald, Dubos François, Leger Pierre-Louis, Berthomieu Lionel, Titomanlio Luigi, Baudin Florent, the Société Française de Pédiatrie (SFP), Linglart Agnès, Gras-Leguen Christèle, Basmaci Romain, Bersani Audrey, Feuillebois Candice, Dutron Sarah, Hage Chehade Mohamad, Lamarcq Mélanie, Lecomte Romain, Loubière Lucie, Tesniere Marc, Moukagni Pelzer Marie, Boussard Noël, Fournier Philippe, Pordes Charlotte, Tamine Muriel, Mathurin Christophe, Ammouche Clément, Ramy Charbel, Maugard Thierry, Chevret Laurent, Jouancastay Mylène, Forgeron Aude, Naud Julien, Aubin Juliette, Demarque Nathalie, Estevez Cyrielle, Desrumaux Amélie, Gatin Amélie, Borsa Dorion Anne, Darviot Estelle, Tronche Julie, Micaelli Xavier, Martinat Laurence, Thibault Marielle, Guilluy Olivier, Laurent Pradeaux, Wyckaert Marine, Veauvy Juven, Bendavid Matthieu, Tchomakov Dimitar, Dupont-Frugier Amélie, Blandine, Chavy Camille, Talal Douksi, Navarro Caroline, Lasserre Claire, Cixous Emmanuel, Le Stradic Camille, Zimmermann Brigitte, Lemoine Lestoquoy Catherine, Brechignac, Kieffer Jerome, Thiriez Gérard, Mirete Justine, Allirand Julien, Groussaud Dominique, Blanchard Olivier, Roux Chrystèle, Marques Cecilia, Bekka Djamek, Limoges Marie Emmanuelle, Boutry Morgane, Micheli Julie, Dupenloup Cecilia, Baret Marie, Babe Philippe, Jean Sandrine, Calbete Maialen, Carle Olivier, Salomez Sophie, Degeorges Marie, Minodier Philippe, Chappuy Helene, Sieng Sorya, Margelidon Sylvie, Pouessel Guillaume, Tronc Frederic, Mediamolle Nicolas, Toulorge David, Barakat Issam, Malterre Aline, Tourteau Laetitia, Naudet Gabrielle, Soussan Banini Valerie, Mahmoudi Sana, Moulin Pierre, Azemar Benjamin, Badier Isabelle, Pellegrino Béatrice, Pailhe Laurence, Mizzi-Rozier Marie, Cannard Margot, Ursulescu Nicoleta, Toma Anne, Lida Ismail, Al Junaidi Ashraf, Belleau Céline, Monlibert Beatrice, Rustom Pecciarini Najla, Armouche Samar, Martinat Laurence, Coulougnon Inès, Favre Lydie, Billaud Nicolas, Horea Cosmina, Brunel Angélique, Binacchi Aurelie, Deville Marie, Saba Lucile, Delfour Antonine, Raimond Florence, Laroui Ahmed, Mery Elodie, Bansept Claire, Royet Olivier, Chomton Cailliez Maryline, Szulc Marie, Razanamparany Voahangy, Le Treust Muriel, Banegas Danielle, Maman Linda, Schneider Fanny, Broenen Emmi, Chazalette Agnes, Chapelon Emeline, Blohm Nathalie, Dolhem Philippe, Racoussot Sylvie, Bengrina Mabrouk, Vanel Noemie, Tanné Corentin, Buisson François, Abdanne Mohamed, Morand Aurelie, Eyer Didier, Rolland Anne, Gaschignard Jean, Diallo Thierno, Gaillard Fanny, Testard Hervé, Bodet Louis, Rakotoharinandrasana-Rason Iarolalao, Meurice Laura, Piloquet Jean-Eudes, Zayat Noura, Bancourt Alice, Dessioux Emmanuelle, Bahnana Joseph, Boulyana Mohamed, Boulyana Mohamed, Boulyana Mohamed, Feudjio Brice, Harchaoui Samir, Coutant Marie, Gariazzo Luisa, Bailly-Bourbigot Mathilde, Lopez Clémence, Prudent Muriel, Losfeld Mathilde, Herve Thierry, Koudsi Mounzer, Barutwanayo, Mazeghrane, Ponthier Laure, Gufflet Marie, Vignaud Olivier, Percheron Lucas, Moulene Eric, Ponah Heng, Roche Christine, Boudes Delphine, Maciow Benjamin, Georges Anne Sophie, Tran Duc-Minh, Rebelle Charlotte, Mirela Dumitrescu, Martinot Justine

**Affiliations:** a ULR 2694 – METRICS : Évaluation des technologies de santé et des pratiques médicales, Univ. Lille, CHU Lille, Lille, France; b French National Out-of-Hospital Cardiac Arrest Registry Research Group – Registre électronique des Arrêts Cardiaques, Lille, France

**Keywords:** Pediatric in-hospital cardiac arrest, Pediatric life-threatening emergencies, Guideline adherence, Advanced life support training, Resuscitation team organization, Quality improvement in pediatric resuscitation

## Abstract

**Background:**

Pediatric in-hospital cardiac arrest (pIHCA) and pediatric in-hospital life-threatening emergencies (pIHLTE) are rare but require rapid, coordinated, guideline-based responses. Little is known about hospitals organization and adherence to international recommendations for these situations. This study evaluated practices regarding the prevention, management, and follow-up of pIHLTE and pIHCA.

**Methods:**

A nationwide cross-sectional survey was sent to all French hospitals providing pediatric care (November 2023–December 2024). Questionnaire assessed adherence to 15 recommendations from European Resuscitation Council guidelines, covering prevention, treatment, and post-event processes. Analyses were conducted at hospital level.

**Results:**

Among 263 eligible hospitals, 181 (68.8%) responded. Adherence to individual recommendations ranged from 25% to 100%. All hospitals reported an emergency call system, an pediatric advanced life support (PALS) response, and personnel trained in advanced pediatric cardiopulmonary resuscitation (CPR). Hospitals followed a median of 10/15 recommendations (IQR 7–12), and 36% met more than 11. Dedicated PALS teams existed in 35% centers; response times <3 min concerned 41%. Training remained inconsistent, with only 48% offering comprehensive Pediatric Basic Life Support (PBLS)/PALS programs. Debriefing practices varied: 60% conducted them routinely, 25% performed none. Barriers included time constraints (65%), medical (52%) or paramedical (48%) staffing shortages.

**Conclusion:**

This nationwide study identifies key gaps in alignment with European guidelines for pIHCA and pIHLTE in France. Standardized emergency call numbers, minimum PALS certification coverage, and mandated debriefing and audit processes might improve care quality and consistency.

## Highlights

• Pediatric in-hospital cardiac arrest is rare, and European epidemiological data remain limited despite ERC/AHA guidelines.
• In 181 French hospitals, median adherence to 15 international pediatric emergency recommendations was 55%.
• Gaps involve prevention systems, standardized protocols, pediatric ALS teams, training and debriefing.
• Only 35% had dedicated pediatric ALS team, and fewer than a half ensured guideline-level BLS/ALS training.
• Staffing shortages and lack of protected time hinder implementation; stronger preparedness and harmonized training are needed.

## Introduction

Pediatric in-hospital cardiac arrests (pIHCAs) and pediatric in-hospital life-threatening emergencies (pIHLTEs) are relatively rare compared with adult events.^1^, ^2^, ^3^ In the United States, pIHCAs occur at a rate of 0.77 per 1,000 hospital admissions, affecting approximately 15,000 children annually.^3^, ^4^ Despite their critical nature, epidemiological data on pIHCAs and pIHLTEs remain limited. In Europe, for example, only six countries maintain registries that include both in-hospital and out-of-hospital cardiac arrests in children, and only three collect pediatric-specific data.^5^ Nevertheless, robust European and American guidelines have been established for the management of these rare but high-stakes situations.^6^, ^7^, ^8^, ^9^

Effective in-hospital cardiopulmonary resuscitation (CPR) is strongly associated with well-defined and appropriately structured organizational frameworks, including clearly assigned roles and responsibilities within multidisciplinary teams.^10^, ^11^, ^12^ A recent U.S. literature review on pIHCAs underscored the substantial gaps in available data but highlighted several key determinants of improved long-term outcomes, such as early risk identification, high-quality CPR, and optimal post-return of spontaneous circulation (ROSC) intensive care.^1^

In addition, preventive strategies, well-trained and well-structured response teams, systematic and structured debriefings, and routine data collection have been identified as essential components for enhancing the management and outcomes of pediatric emergencies.^13^, ^14^, ^15^, ^16^ Regular training of medical personnel in pediatric basic and advanced life support is recommended, and pediatric hospitals are encouraged to align their practices with international guidelines for pIHCAs and pIHLTEs.^17^, ^18^

To date, no studies have examined the organizational structure of response teams or the strategies used to manage pIHLTEs and pIHCAs in pediatric hospitals, nor have any assessed the degree to which current practices adhere to international recommendations.^6^, ^7^ Therefore, the primary objective of this study was to conduct a comprehensive evaluation of existing protocols and operational practices across pediatric hospitals nationwide, with a focus on adherence to international recommendations for the management of pIHLTEs and pIHCAs. Secondary objectives were to identify key barriers to the implementation and long-term sustainability of these protocols and recommendations.

## Methods

### Identification of international recommendations

The recommendations considered in this study addressed key organizational components, including institutional alert systems, the availability of a dedicated emergency number, staff mobilization and training, standardized response protocols, deployment of emergency equipment, and staff awareness and compliance with established procedures.^11^ A total of 15 recommendations from the European Resuscitation Council (ERC) 2021, aligned with AHA guidance through the ILCOR consensus, relevant to the management of pIHCA and pIHLTE were identified.^8^, ^9^ There were no specific pediatric recommendations.

Seven recommendations focused on prevention:

R1 – Clear policy for the clinical response to abnormal vital signs and critical illness
R2 – Early warning score system for the timely identification of critically ill patients or those at risk of deterioration
R3 – Training staff in recognition, monitoring and immediate management of critically ill patients
R4 – Empowerment of all staff to call for help when a patient at risk of deterioration is identified
R5 – Use of structured communication tools
R6 – Systematic review of pIHCA or pIHLTE events to improve practice and share learning points
R7 – Availability of standardized hospital emergency number for cardiac arrest (CA) calls (2222)

Eight recommendations focused on treatment:

R8 – Hospital systems enabling prompt recognition of CA, initiation of CPR and rapid defibrillation (<3 min)
R9 – Trained staff in recognition, activation of the response system and rapid defibrillation
R10 – Immediate response by a designated resuscitation team in cases of pIHCA or pIHLTE
R11 – Resuscitation team members certified in accredited Pediatric Advanced Life Support (PALS) courses
R12 – Resuscitation team competence in advanced CPR management (manual defibrillation, advanced airways management, intravenous access, intra-osseous access, identification and treatment of reversible causes)
R13 – Clear allocation of roles within the resuscitation team
R14 – Standardization of resuscitation equipment across the institution

One recommendation concerned post-event follow-up:

R15 – Performance-focused debriefing of rescuers to improve CPR quality and patient outcomes

Each recommendation was classified as followed (partially if more than half of the staff were able to comply or fully if all staff members were able to comply) or not followed. The total number of recommendations met by each hospital was quantified and analyzed.

### Hospital recruitment

All 13 regions of mainland France (mean population share 7.7%), excluding Corsica (maritime region, 0.5% of France’s population), were represented. Based on publicly available data from the French Hospital Federation, a comprehensive list of hospitals with pediatric activity in mainland France was established. All hospitals providing pediatric care were eligible; hospitals without pediatric services were excluded.

Pediatric intensivists were contacted through the Groupe Francophone de Réanimation et d’Urgences Pédiatriques (GFRUP) and the Pediatric Intensive Care Unit Registry (PICURe – RéAC). Hospital-based pediatricians were contacted via the French Society of Pediatrics (SFP).

### Reporting guidelines

This web-based survey was conducted in accordance with the Checklist for Reporting Results of Internet E-Surveys (CHERRIES) guidelines.^19^

### Study design and development

A national online survey on pIHCAs and pIHLTEs was distributed to all hospitals in France providing pediatric care between November 2023 and December 2024. The structured questionnaire assessed organizational models, management strategies, and team structures related to the management of pIHLTEs and pIHCAs. Ethical approval was not required, as no patient data were collected.

The survey was developed in French using the Framaforms® platform. Pilot testing was carried out among pediatric intensivists at Lille University Hospital to assess clarity, feasibility, and content validity.

### Survey description

The final questionnaire included 41 questions, grouped into ten sections (Supplemental Fig. 1):

1. Hospital and respondent characteristics
2. Organization of pIHLTE management
3. Pediatric Basic life support (PBLS; e.g., bag-valve-mask ventilation, chest compressions)
4. Pediatric Advanced life support (PALS; e.g., defibrillation, epinephrine use)
5. ALS team composition
6. Team roles during PALS
7. Required resuscitation equipment
8. Training policies
9. pIHCA management
10. Barriers to implementation and sustainability of pIHLTE protocols

### Survey distribution

After validation, the survey was disseminated by email to all hospital departments providing pediatric care, as well as directly to physicians when contact information was available (pediatricians, emergency physicians, pediatric intensivists, anesthesiologists, etc.). A structured follow-up strategy was used for non-respondents: an initial reminder email, telephone contact via department secretaries, and a final reminder email.

### Statistical analysis

Analyses were conducted at the hospital level. Responses extracted from the Framaforms® platform were screened for completeness; incomplete or ineligible submissions were excluded. Very few hospitals submitted multiple responses (<5%). In such cases, one response per hospital was retained. A conservative strategy was applied by selecting the response with the lowest scores to reduce the risk of overestimation.

Data were analyzed using IBM SPSS Statistics (version 22.0; IBM Corp., Armonk, NY, USA). Categorical variables are presented as counts and percentages, and continuous variables as medians with interquartile ranges (IQRs).

## Results

### Hospital characteristics and survey respondents

**Fig. 1:**
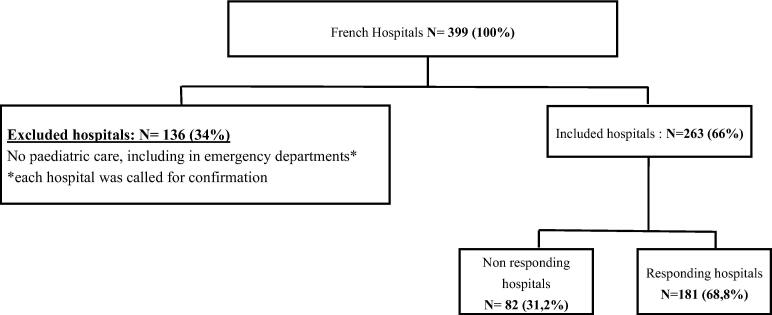
**Study flow-chart**.

Of the 399 hospitals initially identified, 263 met the inclusion criteria, and 181 (68.8%) completed the survey (Fig. 1). Organizational differences were observed between responding and non-responding hospitals. Responding hospitals were more likely to have pediatric intermediate care units (23.8% vs. 6.1%, *p* = 0.001), pediatric intensive care units (14.9% vs. 2.4%, *p* = 0.003), and neonatal intensive care units (26.5% vs. 3.7%, *p* < 0.001) (Table 1). Among the 181 responding hospitals, 68.6% had pediatric emergency departments, 91.7% had pediatric intermediate units, and 14.9% had pediatric intensive care units. Most respondents were pediatricians (63%) (Supplemental Fig. 1).

**Table 1:** Hospitals characteristic.

	**Included hospitals** ***N* = 263**	**Responding hospitals** ***N* = 181 (68.8)**	**Non-responding hospitals** ***N* = 82 (31.2)**	***p***
Pediatric emergencies, *n* (%)	158 (60.1)	115 (63.5)	43 (52.4)	0.089
Pediatric conventional care unit, *n* (%)	242 (92)	166 (91.7)	76 (92.6)	0.788
Pediatric continuous care unit, *n* (%)	48 (18.3)	43 (23.8)	5 (6.1)	**0.001**
Pediatric intensive care unit, *n* (%)	29 (11)	27 (14.9)	2 (2.4)	**0.003**
Neonatal intensive care unit, *n* (%)	51 (19.4)	48 (26.5)		**<0.001**
French Regions, *n* (%)				0.409
Auvergne-Rhône-Alpes	33 (12.5)	26 (14.4)	7 (8.5)	
Bourgogne-Franche-Comté	15 (5.7)	12 (6.6)	3 (3.7)	
Bretagne	14 (5.3)	9 (5)	5 (6.1)	
Centre Val de Loire	12 (4.6)	7 (3.9)	5 (6.1)	
Grand Est	28 (10.6)	18 (9.9)	10 (12.1)	
Hauts de France	29 (11)	21 (11.6)	8 (9.8)	
Ile de France	35 (13.3)	21 (11.6)	14 (17)	
Normandie	19 (7.2)	11 (6.1)	8 (9.8)	
Nouvelle Aquitaine	25 (9.5)	14 (7.7)	11 (13.4)	
Occitanie	22 (8.4)	19 (10.5)	3 (3.7)	
Pays de la Loire	12 (4.6)	9 (5)	3 (3.7)	
Provence Alpes Côte d’Azur	19 (7.2)	14 (7.7)	5 (6.1)	

### Prevention of pIHCAs and pIHLTEs

All recommendations and the proportion of hospitals following them are presented in Table 2.

**Table 2:** Hospitals responses to international guidelines.

**Prevention of pIHCA and pIHLTE**	**N/Effectif**	**(%)**
R1 – Clear policy for the clinical response to abnormal vital signs and critical illness	**129/181**	**(71)**
R2 – Early warning score systems for the timely identification of critically ill patients*	**129/181**	**(71)**
– Posted on walls	88/129	(68)
– Displayed on emergency carts	77/129	(30)
– Available on smartphone	50/129	(39)
– Communicated orally	33/129	(26)
– Communicated via email	25/129	(19)
– Not communicated	12/129	(9)
R3 – Training staff in recognition, monitoring and immediate management of critically ill patients	**156/181**	**(86)**
– All staff**	81/181	(45)
– Some**	75/181	(41)
– No one	24/181	(13)
R4 – Empower all staff to call for help when they identify a patient at risk of physiological deterioration	**89/181**	**(49)**
R5 – Use of structured communication tools (Written algorithm)	**132/181**	**(73)**
R6 – Systematic review of pIHCA or pIHLTE events to improve practice and share learning points	**45/181**	**(25)**
– Structured monitoring tool	29/181	(16)
R7 – Availability of standardized hospital emergency number for cardiac arrest calls (2222)*	**181/181**	**(100)**
– 2222	11/181	(6)
– Another number than 2222	146/181	(81)
– Several phone numbers without 2222	6/181	(5)
– Several phone numbers with 2222	11/181	(6)
– Daily changing phone number	10/181	(6)

**Treatment of pIHCA and pIHLTE**		
R8 – Hospital systems enabling prompt recognition of cardiac arrest recognition, initiation of CPR and rapid defibrillation (< 3 min)	**139/181**	**(77)**
– Full staff trained**	17/181	(9)
– > than 50 of the staff trained**	122/181	(67)
– < than 50 of the staff trained	36/181	(20)
R9 – Trained staff in recognition, activation of the response system and rapid defibrillation (PBLS)	**88/181**	**(49)**
– Simulation center accessibility	108/181	(60)
R10 – Immediate response by a designated resuscitation team in cases of pIHCA or pIHLTE	**181/181**	**(100)**
– Pediatric dedicated ALS team	63/181	(35)
R11 – Resuscitation team members certified in accredited Pediatric Advanced Life Support (PALS) courses	**61/181**	**(34)**
R12 – Resuscitation team competence in advanced CPR management (manual defibrillation, advanced airways management, intravenous access, intra-osseous access, identification and treatment of reversible causes)*	**181/181**	**(100)**
– Pediatrician	87/181	(48)
– Pediatric Intensivist	32/181	(18)
– Pediatric emergency doctor	24/181	(13)
– Emergency Medical Service doctor	52/181	(29)
– Adult Intensivist	37/181	(20)
– Anaesthesiologist	35/181	(19)
– Adult emergency doctor	44/181	(24)
R13 – Clear allocation of roles within the resuscitation team	**111/181**	**(61)**
– Team leader designation**	80/181	(44)
– Distributed role just before pIHCA or pIHLTE	14/181	(24)
R14 – Standardization of resuscitation equipment across the institution	**142/181**	**(79)**
– ALS team bag	135/181	(75)

**Follow-up of pIHCA and pIHLTE situations**		
R15 – Performance-focused debriefing of rescuers to improve CPR quality and patient outcomes	**123/181**	**(68)**
– Immediately**	42/181	(23)
– 1–2 weeks**	66/181	(37)
– >1 months**	15/181	(8)

pIHCA: pediatric in hospital cardiac arrest; pIHLTE: pediatric life-threatening emergencies.

* Multiple choice questions.

** Conditions for validation of the recommendation.

#### R1 and R2 – Policies and early warning systems

A clear procedure for identifying abnormal vital signs or critical illness was implemented in 129 hospitals (71%), posted on hospital walls in 68% and attached to emergency carts in 60%. Very few hospitals submitted multiple responses (<5%). In such cases, one response per hospital was retained. A conservative strategy was applied by selecting the response with the lowest scores to reduce the risk of overestimation. Procedures differed between adults and children in 107 hospitals (59%). Twenty-seven hospitals (15%) reported variations between working hours and off-hours.

#### R3 – Staff training

Training in the recognition and immediate management of pIHCAs/pIHLTEs was reported by 156 hospitals (86%), either for all staff (45%) or for selected staff (41%). Training modalities included in-ward simulation (62%), theoretical sessions – focused on theoretical knowledge without practical training – (44%), and/or sessions in dedicated simulation centers (43%).

#### R4 – Empowerment to call for help

Eighty-nine hospitals (49%) had a formal training policy; 64 (35%) offered communication training, and 23 (13%) organized mock alerts.

#### R5 – Structured communication tools

A written algorithm for pIHCA management was available in 132 hospitals (73%), with cognitive aids used in 104 (79%) of these.

#### R6 – Event review

**Fig. 2:**
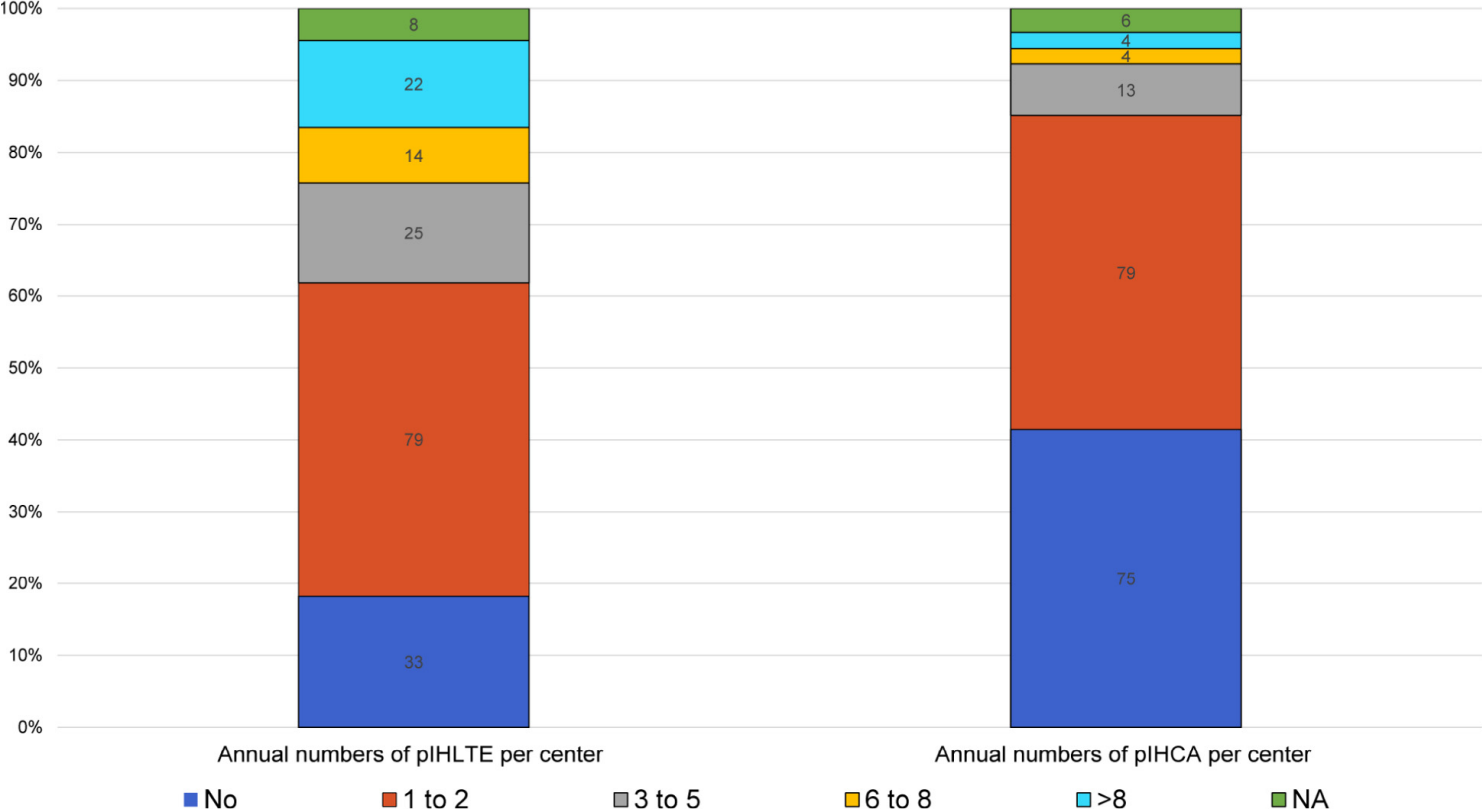
**Number and frequency of pIHCA or pIHLTE events per hospital (Recommendation 6)**. *Abbreviations:* pIHCA, pediatric in-hospital cardiac arrest; pIHLTE, pediatric in-hospital life-threatening emergencies.

Annual monitoring of pIHCA or pIHLTE events occurred in 25% of hospitals. Structured processes (event forms, electronic reporting, or morbidity and mortality reviews) were used in 16%. Annual frequencies of pIHCA and pIHLTE are shown in Fig. 2.

#### R7 – Standardized emergency number

All hospitals reported using a dedicated emergency number. Eleven hospitals (6%) used the standardized 2222 number, whereas 146 (81%) used a specific number for pIHLTE activation.

### Treatment of pIHCAs and pIHLTEs

#### R8 – Prompt recognition, CPR and defibrillation

PBLS could be initiated immediately in 77% of the hospitals (by all staff in 9% of hospitals and by most staff in 67%). The location of the defibrillator was known in 95%.

#### R9 – PBLS trained staff

Forty-eight percent of hospitals had ERC-standard PBLS teams; 60% had access to a simulation center.

#### R10 – Immediate PALS response

All hospitals reported the presence of an PALS team. A dedicated PALS team was available in 35% of hospitals. Response times were <3 min in 41% and 3–6 min in 49%. Team size ranged from one to three members in 54% of hospitals and exceeded three members in 21%.

#### R11 – PALS certification

Formal ALS course certification was present in 34% of hospitals. However, the ERC algorithm was used in 72%.

#### R12 – Advanced CPR competence

All hospitals (181, 100%) reported having at least one physician with advanced CPR skills (advanced airway management, vascular access, administration of resuscitation medication, cardiac rhythm recognition).^6^ Events were managed by a pediatrician (48%), a pediatric intensivist (18%), or a pediatric emergency physician (13%). Nurses with PALS skills were present in 49% of hospitals. Students participated in resuscitation events in 67% of hospitals, predominantly residents (97%).

#### R13 – Role allocation

Clear allocation of roles was defined in 61% of hospitals. A designated team leader was present in 44%, and predefined roles were reported in 21%. Among hospitals without predefined roles, 24% assigned roles ad hoc.

#### R14 – Standardized equipment

Emergency carts were available in 98% of hospitals; contents were standardized in 79%, and 75% also had an emergency bag.

### Follow-up of pIHCAs and pIHLTEs

#### R15 – Debriefing

Debriefings were performed within 1–2 weeks in 60% of hospitals, after more than one month in 8%, and not conducted at all in 25%.

**Fig. 3:**
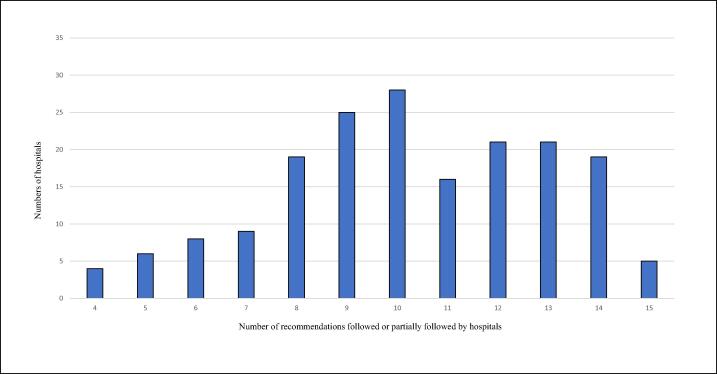
**Recommendations followed by hospitals**.

Across the 181 hospitals, the median number of recommendations followed was 10 [IQR: 7–12], corresponding to 55% adherence. Eighteen hospitals (10%) followed six or fewer recommendations, whereas 66 hospitals (36%) followed more than 11 (Fig. 3).

### Barriers to implementation and sustainability of pIHLTE procedures

Insufficient medical or paramedical staffing hindered the implementation of pIHLTE procedures in 95 (52%) and 87 (48%) hospitals, respectively. The number of physicians available for PALS-team duties ranged from fewer than five in 63 hospitals (35%), five to 10 in 38 hospitals (21%), and more than 10 in 24 hospitals (13%).

**Fig. 4:**
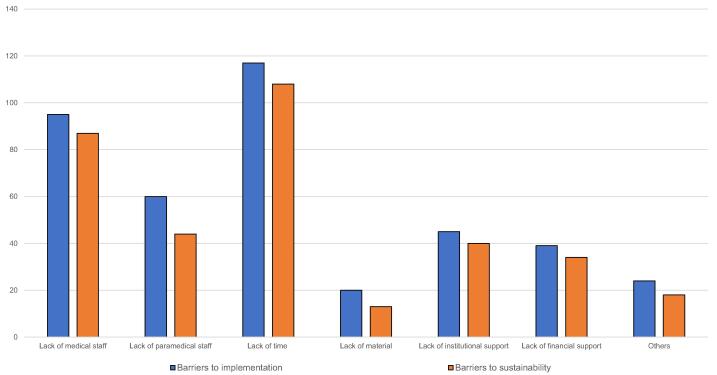
**Barriers to implementation and sustainability of pIHLTE and pIHCA procedures (*N* = 181)**. *Abbreviations*: pIHLTE, pediatric in-hospital life-threatening emergencies; pIHCA, pediatric in-hospital cardiac arrest.

Staffing shortages were also perceived as a barrier to the long-term sustainability of pIHLTE procedures in 60 hospitals (33%) for medical staff and 44 hospitals (24%) for paramedical staff. Lack of time was the most frequently reported barrier to the implementation and sustainability of these procedures (117 hospitals [65%] and 108 hospitals [60%], respectively). Additional barriers, including limited financial resources, are shown in Fig. 4.

## Discussion

This study represents the first national operational audit of pediatric in-hospital emergency preparedness. Based a large-scale survey of 181 hospitals, it provides the first comprehensive overview in France of organizational structures and management protocols for pIHLTE and pIHCA. Across the 15 international recommendations assessed, adherence ranged from 25% to 100%. All hospitals reported having a call procedure (R7), immediate response capability (R10), and a dedicated emergency team (R12). However, only half of the hospitals complied with at least 53% of the recommendations overall. Staffing shortages and time constraints were the main barriers to the effective and sustainable implementation.

European guidelines emphasize rapid recognition of pIHCA and initiation of CPR within 3 min.^8^ Early response is crucial. Sandroni et al. (2004) showed significantly higher survival when the cardiac arrest team arrived within 3 min, and no survival when arrival exceeded 6 min. In our study, 71% of hospitals reported having a dedicated protocol, but protocol visibility and operational were inconsistent. Only 35% of centers had a dedicated PALS team, despite evidence linking such teams to improved outcomes.^20^

Regarding emergency call numbers, only 6% of hospitals specifically used the standardized number 2222, although all had a dedicated number for pIHLTE. Limited implantation of the 2222 system has also been reported across Europe, with only 2% of countries using it.^21^ The use of a standardized emergency number (2222) might represent a potential high-impact, low-cost national improvement lever.

Hospitals with dedicated PALS teams generally met international response-time recommendations, with 90% responding within 5 min.^6^, ^9^ In contrast, hospitals without such teams relied on ad hoc mobilization, contributing to variability in care. This variability seems clinically significant as response times of more than 3 min have been associated with markedly reduced survival outcomes.^20^ Strengthening pediatric emergency training for adult emergency teams may help reduce this variability, particularly in mixed-care settings.^15^

The ERC algorithm was widely used (72%). However, only 34% of centers reported formal PALS certification and only 48% provided guideline-level PBLS/PALS training. This discrepancy suggests algorithm compliance without corresponding training compliance.^11^, ^12^ A lack of protected time was frequently cited as a barrier to implementing and sustaining pILTHE procedures.

Team composition also varied considerably. Pediatric intensivists were not systematically involved, especially in non-university hospitals, consistent with previous reports.^11^ Many hospitals reported insufficient leadership and unclear role allocation during emergencies, despite strong evidence supporting the importance of effective team leadership.^11^, ^22^, ^23^, ^24^ Debriefing practices were similarly inconsistent. While 60% of hospitals conducted debriefings, one-quarter reported none, limiting opportunities for learning and quality improvement.^15^, ^16^, ^25^

Standardized protocols are central to effective pIHLTE management.^15^, ^26^ In this survey, only 71% of hospitals had such protocols in place. This lack of standardization is particularly concerning in time-critical emergencies, where procedural clarity directly impacts survival.^20^, ^27^, ^28^

Regarding training (R9), only 48% of hospitals reported that all pediatric staff had received adequate training in recognizing and managing pediatric cardiac arrest. Training policies are known to influence survival after pIHCA and pIHLTE.^17^, ^29^ In-situ simulation was the most commonly used educational method and is well established as effective.^18^, ^30^ However, delivering PBLS and PALS training at guideline-recommended levels (R11) remains challenging and requires substantial resources and procedural standardization.^31^ These findings highlight the need for institutional support, protected training time, improved access to simulation, and national-level coordination to ensure consistent training practices.^32^ Successful national initiatives in neighboring countries should encourage similar developments in France.^33^

Those findings support the development of a national – and potentially international- framework along with regular audits, to drive continuous quality improvement in the management of pIHLTE.^15^, ^34^

This study is based on recommendations derived from the 2021 European Resuscitation Council guidelines, which are consistent with American Heart Association guidance through the ILCOR consensus process. In addition, the 2025 guidelines further emphasize early defibrillation, team performance, system standardization, regular training including simulation, and effective leadership during resuscitation. The recommendations from 2021 have been maintained in the 2025 resuscitation guidelines. In addition, 2025 ERC guidelines introduce further organizational guidance and incorporate pediatric-specific concepts, thereby reinforcing the continued relevance of our findings.^6^, ^7^, ^8^, ^9^, ^21^, ^35^

### Strengths

This is the first nationwide study in France to evaluate adherence to international guidelines for pIHLTE and pIHCA management. The high response rate (68.8%) among eligible hospitals strengthens the reliability of the findings, and the inclusion of almost all French regions enhances generalizability.^36^ The survey captured detailed information on organizational structures, system preparedness, and training practices. Importantly, the individualized assessment of each hospital allows for targeted feedback to support local improvement efforts. The use of a structured survey allowed for reproducible and comprehensive data collection. Data analysis with an individual assessment of each hospital will enable us to provide specific feedback to each hospital for personalized awareness of strengths and weaknesses in adherence to the recommendations of the Intra-hospital Life-Threatening Emergencies for each hospital.

### Limitations

This study has several limitations. First, it was restricted to French pediatric hospitals and did not include data from other European countries. Expanding the survey to a European cohort proved challenging due to the absence of a centralized contact registry for eligible pediatric centers. Second, despite four reminders, a 100% response rate could not be achieved. Some structural differences were observed between responding and non-responding hospitals, with responding hospitals more frequently reporting pediatric-specific facilities (pediatric continuous care units, pediatric intensive care units, and neonatal intensive care units). These differences may have influenced the representativeness of the findings, as such hospitals tend to be larger and more likely to have staff with pediatric expertise.^10^ Finally, the reliance on self-reported data may have introduced reporting bias, although the structured CHERRIES-compliant design aimed to minimize this risk.

## Conclusions

This nationwide study provides a comprehensive overview of the management of pIHCA and pIHLTE across French hospitals, and highlights key gaps in alignment with European guidelines.^6^ Addressing these gaps requires targeted system-level measures, including standardization of the in-hospital emergency call number, definition of minimum PALS certification coverage, and mandated post-event debriefing and audit loops.

## CRediT authorship contribution statement

**Marguerite Lockhart-Bouron:** Writing – review & editing, Writing – original draft, Validation, Project administration, Methodology, Formal analysis, Data curation, Conceptualization. **Johann Exbrayat:** Writing – review & editing, Methodology, Formal analysis, Data curation. **Valentine Baert:** Writing – review & editing. **Hervé Hubert:** Writing – review & editing. **Morgan Recher:** Writing – review & editing, Methodology, Conceptualization. **Stéphane Leteurtre:** Writing – review & editing, Methodology, Conceptualization.

## Declaration of competing interest

The authors report no conflicts of interest. The authors alone are responsible for the content and writing of the paper.
