# Supplementary material for: Pediatric in-hospital life-threatening emergencies and cardiac arrest in France: adherence to international guidelines and barriers to implementation
Source: Resusc Plus. 2026 Feb 10;28:101261. doi: 10.1016/j.resplu.2026.101261 (PMC12937156; doi:10.1016/j.resplu.2026.101261)
Supplement: Supplementary Data 1 [file mmc1.docx]

***Supplemental Figure 1.***

**French National Web-Survey**

**Relevance of International Guidelines and Barriers in the Implemention of the “Pediatric In-Hospital Life-Threatening Emergencies and In-Hospital Cardiac Arrest” procedure**

1. **Characteristics of the Institution and the Respondent:**

A.1. Country:

A.2. City where the institution is located:

A.3. Type of hospital: ☐ University Hospital ☐ Hospital Centre ☐ Other

A.4. Department where the respondent works:
☐ Pediatric Emergency Unit
☐ Pediatric Continuous Care Unit
☐ Pediatric Intensive Care Unit
☐ General Pediatric Ward
☐ General Emergency Department
☐ Other

A.5. Profession of the respondent:

☐ Pediatrician
☐ Pediatric Emergency Physician
☐ Pediatric Anesthesiologist
☐ Neonatologist
☐ Pediatric Intensivist
☐ Adult Emergency Physician
☐ Adult Anesthesiologist or Intensivist
☐ Other

1. **Organization of In-Hospital Life-Threatening Emergencies (IHLTE) in Your Facility (Excluding Pediatric Intensive Care Units)**

B.1. Is there a clearly defined procedure for pediatric IHLTE in your hospital? ☐ Yes ☐ No

B.1bis. If yes, how is the IHLTE call/alert procedure communicated?

☐ Posted on walls
☐ Displayed on emergency carts
☐ Available on telephones
☐ Communicated orally
☐ Communicated via email or internal mail
☐ Other
☐ Not communicated

B.2. Is there a different call/alert procedure?

B.2.a. Between adult and pediatric IHLTE?  YES  NO

B.2.b. Between working and off-hours?  YES  NO

B.3. What emergency number(s) are used for IHLTE in your hospital?

☐ 2222 only
☐ Another number
☐ Multiple numbers (excluding 2222)
☐ Multiple numbers including 2222 (number changes daily)

B.4. Is the annual number of pediatric IHLTE activations monitored by hospital management or the medical director? YES  NO

B.5. 3.On average, how many times per year is the pediatric IHLTH procedure triggered for the following situations?

- 1. Pediatric IHLTE situations :  0 1 à 2 3 à 5 6 à 8 > 8
  2. In-hospital pediatric cardiopulmonary arrest  0  1 à 2  3 à 5  6 à 8  > 8
  3. Is there a monitoring or follow-up tool in place?  YES  NO

1. **Basic Life Support (BLS = Bag-valve-mask ventilation + Chest compressions)**

C.1. In your hospital, are staff in pediatric wards specifically trained to promptly recognize pediatric cardiac arrest?

☐ YES, in all departments
☐ YES, in selected departments only
☐ NO, in none of the departments

C.1bis. If yes, how is this training delivered?

☐ Paper-based cognitive aids or manuals
☐ In-situ simulation training
☐ On-site theoretical training sessions
☐ Training at a dedicated simulation center
☐ Other (please specify): ___________

C.2. What proportion of staff in pediatric departments are capable of initiating basic cardiopulmonary resuscitation immediately?

☐ None
☐ 1 or 2 individuals
☐ Less than half of the team
☐ About half of the team
☐ More than half of the team
☐ Almost the entire team
☐ The entire team

1. **Advanced Life Support (e.g., Shock, Epinephrine, Intraosseous Access)**

D.1. Does your hospital have a dedicated and identifiable pediatric advanced life support team for in-hospital cardiac arrest? ☐ YES ☐ NO

D.1bis. If yes, what is the average response time for this team to arrive on the scene?
☐ Less than 3 minutes ☐ 3–5 minutes ☐ 6–8 minutes ☐ More than 8 minutes

1. **Composition of the Advanced Life Support Team**

E.1. How many physicians are included in the dedicated ALS team pool?
☐ Fewer than 5 ☐ Between 5 and 10 ☐ More than 10

E.2. On average, how many team members are involved during an advanced resuscitation?
☐ 1 ☐ 2 ☐ 3 ☐ 4 ☐ 5 ☐ More than 5

E.3. What types of personnel compose the dedicated Advanced Life Support team?
☐ Pediatric Intensivist ☐ Adult Intensivist ☐ Anesthesiologist ☐ Nurse ☐ EMS physician ☐ Adult emergency physician ☐ Pediatric emergency physician ☐ Pediatrician ☐ Intern/Resident ☐ Other

E.4. Are students present during advanced life support interventions?
☐ Never ☐ Sometimes ☐ Always

E.4bis. If "Always" or "Sometimes", please specify the type(s) of students involved:
☐ Third-cycle (Interns/Residents) ☐ Second-cycle (Medical students/Externals) ☐ Paramedical (Nursing students)

E.5. In the absence of a dedicated ALS team in your hospital, what type of backup is called upon in the event of an in-hospital pediatric cardiac arrest?
☐ EMS call ☐ On-site EMS physician ☐ Resuscitation team ☐ Pediatric Intensivist ☐ Neonatologist ☐ Adult Intensivist ☐ Anesthesiologist ☐ Adult emergency physician ☐ Pediatric emergency physician ☐ Pediatrician ☐ Intern/Resident ☐ Nurse ☐ Other ☐ None

1. **Team Roles During Advanced Life Support**

F.1. Are the roles of each member of the advanced life support (ALS) team clearly defined in advance?
☐ Always
☐ Sometimes
☐ No
☐ Don't know

F.1.1bis. If No, does the resuscitation team take time to rapidly assign roles within the first few minutes of in-hospital cardiac arrest management?

Always

Sometimes

No

Don't know

F.2. Is a team leader consistently designated?

Always

Sometimes

No

Don't know

F.2bis. If Yes, is the team leader easily identifiable by a specific marker?
☐ No
☐ Initial verbal announcement
☐ Visible distinguishing sign

☐ Unknown

F.3. Is the situation systematically debriefed after the intervention?
☐ No
☐ Immediately
☐ Within 1–2 weeks
☐ More than 1 month later

1. **Equipment required for the management of pediatric in-hospital cardiac arrest**

G.1. Does the dedicated resuscitation team have an emergency bag with equipment?  Yes  No

G.2. Are there emergency carts on the wards?  Yes  No

G.2bis. If yes, are they standardized (identical) throughout the hospital?  Yes  No

G.3. Is the location of emergency carts clearly defined?  Yes  No

G.4. Using your procedure, do service personnel know how to find the manual defibrillator or AED?  Yes  No

1. **Training for childcare teams**

H.1. Does your hospital do unscheduled mock "cardiac arrest" alerts to train teams?  Yes  No

H.2. Does your hospital have a training policy for the management of pediatric in-hospital cardiac arrest for?

H.2bis.a. Basic life support  Yes  No
H.2bis.b. Advanced life support  Yes  No
H.2bis.c. No training

H.3. Do you have access to a simulation centre to train in basic and advanced resuscitation for the management of in-hospital cardiac arrest?  Yes  No

H.4. Do you offer training in communication management during in-hospital cardiac arrest?  Yes  No

1. **Management of pediatric in-hospital cardiac arrest**

I.1. Do you have a written algorithm for the management of pediatric in-hospital cardiac arrest?  Yes  No

I.1bis. If yes, do you have emergency cognitive aids in your wards or on emergency carts?  Yes  No

I.2. Is this algorithm for the management of cardiac arrest of the "European Resuscitation Council" type?  Yes  No

1. **Brakes**

J.1. Have you identified any obstacles to **implementing** the pediatric UVIH procedure? lack of medical staff  lack of paramedical staff  lack of time for organizational set-up  lack of equipment  lack of institutional support  lack of financial resources  other: ...

J.2. Have you identified any obstacles to **making** this procedure **permanent**?  Lack of medical staff  lack of paramedical staff  lack of time for organizational set-up  lack of equipment  lack of institutional support  lack of financial resources  other ......
