# Supplementary material for: Pediatric in-hospital life-threatening emergencies and cardiac arrest in France: adherence to international guidelines and barriers to implementation
Source: Resusc Plus. 2026 Feb 10;28:101261. doi: 10.1016/j.resplu.2026.101261 (PMC12937156; doi:10.1016/j.resplu.2026.101261)
Supplement: Supplementary Fig. 1 [file mmc2.pptx]

## Slide 1
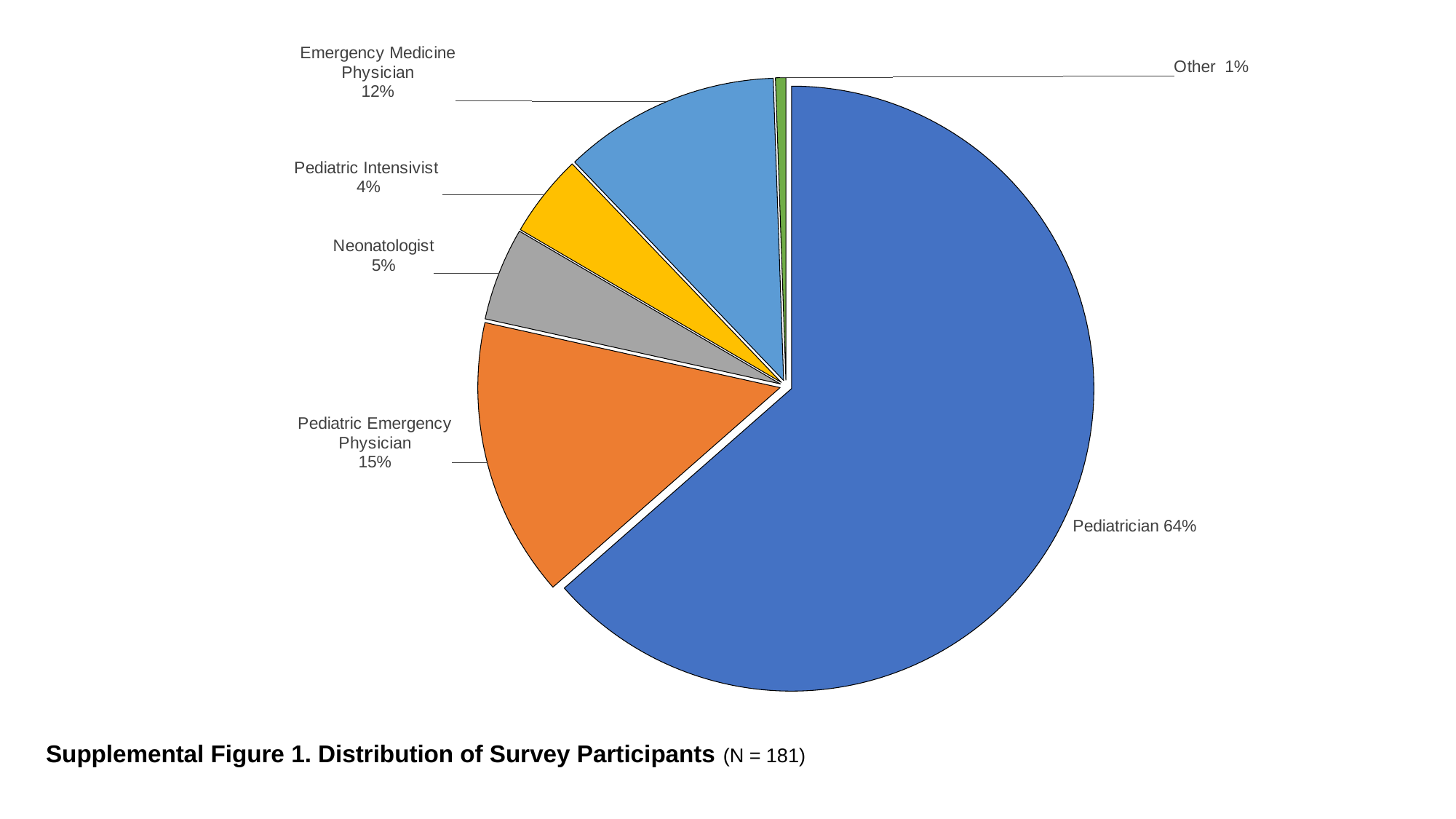

### Chart
| Category | |
|---|---|
| Pediatrician | 0.6353591160220995 |
| Pediatric Emergency Physician | 0.14917127071823205 |
| Neonatologist | 0.049723756906077346 |
| Pediatric Intensivist | 0.04419889502762431 |
| Emergency Medicine Physician | 0.11602209944751381 |
| Other | 0.0055248618784530384 |Supplemental Figure 1. Distribution of Survey Participants (N = 181)
